# Which Dermatological Conditions Present to an Emergency Department in Australia?

**DOI:** 10.1155/2014/463026

**Published:** 2014-03-31

**Authors:** Julia Lai-Kwon, Tracey J. Weiland, Alvin H. Chong, George A. Jelinek

**Affiliations:** ^1^Department of Medicine (Dermatology), St. Vincent's Hospital, Melbourne, VIC, Australia; ^2^Emergency Practice Innovation Centre, St. Vincent's Hospital, 41 Victoria Parade Fitzroy, Melbourne, VIC 3065, Australia; ^3^Faculty of Medicine Dentistry and Health Sciences, The University of Melbourne, VIC, Australia

## Abstract

*Background/Objectives*. There is minimal data available on the types of dermatological conditions which present to tertiary emergency departments (ED). We analysed demographic and clinical features of dermatological presentations to an Australian adult ED. *Methods*. The St. Vincent's Hospital Melbourne (SVHM) ED database was searched for dermatological presentations between 1 January 2009 and 31 December 2011 by keywords and ICD-10 diagnosis codes. The lists were merged, and the ICD-10 codes were grouped into 55 categories for analysis. Demographic and clinical data for these presentations were then analysed. *Results*. 123 345 people presented to SVHM ED during the 3-year period. 4817 (3.9%) presented for a primarily dermatological complaint. The most common conditions by ICD-10 diagnosis code were cellulitis (*n* = 1741, 36.1%), allergy with skin involvement (*n* = 939, 19.5%), boils/furuncles/pilonidal sinuses (*n* = 526, 11.1%), eczema/dermatitis (*n* = 274, 5.7%), and varicella zoster infection (*n* = 161, 3.3%). *Conclusion*. The burden of dermatological disease presenting to ED is small but not insignificant. This information may assist in designing dermatological curricula for hospital clinicians and specialty training organisations as well as informing the allocation of dermatological resources to ED.

## 1. Introduction

Dermatology is primarily an outpatient specialty in Australia. However, up to 5–8% of all emergency department (ED) presentations may be due to dermatological complaints [1]. Despite this, there have been few studies that have characterised the types of patients who present to ED with dermatological conditions, with the most focused on paediatric rather than adult ED presentations [2–4]. The variability in the types of presentations between countries has also been described previously, highlighting the importance of country-specific data [5]. However, Australian data is limited, with only one retrospective study from South Australia found on a review of the literature [6]. We conducted a three-year retrospective study on patients with dermatological conditions presenting to the St. Vincent's Hospital ED in Melbourne, Australia, in order to gather demographic and clinical data on patients who present, and the types of dermatological conditions with which they present. This may better inform Junior Medical Officers and ED physicians of dermatology curriculum design as well as assist with dermatological resource allocation to ED.

## 2. Methods

### 2.1. Data Selection

St. Vincent's Hospital Melbourne (SVHM) ED is an adult tertiary facility which sees approximately 40,000 patients per year. The hospital is situated on the fringe of the city's Central Business District and serves a culturally diverse and socially disadvantaged population. All ED visits between 1 January 2009 and 31 December 2011 were extracted from the hospital database. A keyword search was performed in the “Presenting Complaint” and “Triage Notes” fields of the Victorian Emergency Minimum Dataset (VEMD) at St. Vincent's Hospital Melbourne, as well as an ICD-10 (International Statistical Classification of Diseases, 10th Revision) diagnosis code search in the primary, secondary, and tertiary diagnostic fields in order to identify dermatological presentations. Keywords and ED ICD-10 diagnoses were selected by consultation between researchers (see Appendices A and B in the Supplementary Material). The ICD-10 codes were selected from a list of available ICD-10 diagnoses in the ED and therefore did not include all possible dermatological diagnoses as listed in the full ICD-10 manual. In cases where there was any doubt as to whether the presentation was primarily a skin complaint, the cases were reviewed by the consultant dermatologist (AC). These lists were then merged, and traumatic skin injuries, burns (excluding sunburn), postoperative wound complications, and repeat presentations were excluded. In order to handle the data more efficiently, the 91 ICD-10 codes were grouped into 55 diagnostically related categories. The grouping of diagnoses was determined by consensus between researchers. The demographic and clinical data for these presentations were then analysed.

### 2.2. Data Analysis

Epidemiological data was summarised using frequencies and percentages for nominal data and means and interquartile ranges (IQR) for continuous data using Microsoft Excel 2010. Inferential comparisons of continuous data were made using the Mann-Whitney *U* test, and comparison of categorical data was made using Pearson's chi-squared test.

This study received approval from the Hospital Research Ethics Committee at SVHM (LRR 146/12, 10/10/2012).

## 3. Results

Over the 3-year period, there were 123 345 presentations to the ED. Of these, 4,817 had a primarily dermatological complaint (3.9%). The majority (52.9%) were male, and 7 patients did not have their gender recorded (Table 1).

The most common skin complaints by ICD-10 diagnosis code were cellulitis (*n* = 1741, 36.1%), allergy with skin involvement (*n* = 939, 19.5%), boils/furuncles/pilonidal sinuses (*n* = 526, 11.1%), eczema/dermatitis (*n* = 274, 5.7%), and varicella zoster infections (*n* = 161, 3.3%) (Table 2).

## 4. Discussion

A significant proportion of ED presentations (3.9%) is due to skin complaints, which is slightly higher than previously reported [5–8]. This may be due to our broad definition of dermatological complaints, which included conditions such as cellulitis and boils/furuncles/pilonidal sinuses which are often excluded as they are typically managed by General Medical or Surgical units.

Patients who presented with skin complaints were generally younger than other patients presenting to ED and their presentations were less urgent, with around two-thirds of dermatological patients triaged as category 4 and the second least urgent category in the five-category triage scale used in a number of countries. This was higher than nondermatological ED presentations, where less than half of the patients were category 4. All six category 1 dermatological patients presented with anaphylaxis with a component of skin involvement such as urticaria or angioedema. This highlights the fact that the majority of skin complaints that present to ED do not require immediate emergency care and could often be managed in outpatient settings. This has been previously recognised in the literature [9]. As a result of their lower urgency, patients with skin complaints waited longer to be seen by a doctor than other ED patients.

Patients with a diverse range of skin complaints presented during the three-year period. Cellulitis was the most common condition (36.1%), consistent with previous studies [7, 10]. However, the prevalence of cellulitis may be misleading, as multiple studies have reported high rates of misdiagnosis of cellulitis in ED due the lack of familiarity with more specific dermatological diagnoses such as lipodermatosclerosis and contact dermatitis. In a retrospective study of 196 inpatient dermatology consults [11], a third of patients consulted for cellulitis had a cutaneous mimicker. Similarly, David et al. [12] demonstrated a misdiagnosis rate of 28% for cellulitis cases following review by a Dermatologist and Infectious Diseases specialist. Misdiagnosis of cellulitis may result in inappropriate and costly antibiotic use. In our study, the high rates of cellulitis may also be partly explained by the lower socioeconomic patient population, with a significant proportion of patients being homeless or affected by drugs or alcohol, placing them at higher risk of cellulitis.

A similar study of dermatological presentations to ED was conducted at the Royal Adelaide Hospital in 2011 [6]. Interestingly, the top three diagnoses were similar for both centres, with eczema being the fourth most common diagnosis at St. Vincent's and the fifth most common at the Royal Adelaide Hospital. However, chronic ulcers, which accounted for 3.7% of ED presentations to the Royal Adelaide Hospital, did not feature in the top ten ED diagnoses at St. Vincent's Hospital. This is despite the high prevalence of pyoderma gangrenosum as a discharge diagnosis (13.9% of admissions under Dermatology). On retrospective analysis of their initial ED diagnosis, six pyoderma gangrenosum cases were classified as “nonspecific skin infection,” two as “cellulitis of the lower limb,” two as “nonspecific rash,” and one each as “atopic dermatitis,” “drug rash,” “dermatitis,” “decubitus ulcer,” and “skin and subcutaneous disorder not otherwise specified.” This reflects the difficulty in making definitive dermatological diagnoses in ED due to time restraints and perhaps limited dermatological experience but also the lack of a specific ICD-10 code in ED for chronic ulcerating conditions.

This study may inform the development of evidence-based dermatological curricula for a number of clinician groups involved in ED dermatological assessment and management. It also provides data on the burden of dermatological presentations to ED which may guide resource allocation in this setting. The significant nonspecific diagnosis rate amongst dermatological ED presentations supports the development of a dermatological consulting service in the ED, a concept which has been previously described [7]. Whilst this role is traditionally filled by the on-call dermatological registrar, a dedicated service would be mutually beneficial to both the ED and Dermatology units. Dermatology registrars would receive increased exposure to common initial dermatological presentations, which is often limited in a tertiary hospital environment, as well as provide diagnostic support to their ED colleagues, limiting unnecessary investigations and treatments. However, given that dermatological presentations account for only 4% of ED presentations, such a service may not be an effective use of health care resources.

Other potential options include the use of a teledermatology referral service within the ED. This has been previously studied in Queensland, where a store-and-forward photography system accompanied by relevant history and examination findings was a rapid and accurate diagnostic tool for dermatological presentations to ED [13]. Whilst this study used photos taken with a camera, a Swedish study used multimedia messaging systems (MMS) from mobile phone cameras as an easier way of transmitting information from the bedside, which would be more practical in the ED setting [14]. It was reported that the diagnostic accuracy and adequacy of management decisions made via MMS referrals were similar to store-and-forward teledermatology systems. Such a system could be particularly useful in rural centres where access to dermatology services may be limited, a possibility previously studied in third world countries [15]. However, there are several limitations to an MMS referral system, including poor picture quality, privacy and consent issues surrounding the storage of photos on private cameras, and the inability of a single photo to substitute for a comprehensive skin examination. Hospitals should therefore consider the suitability of teledermatology services for dermatology ED consultations as a means of improving the accuracy of skin-related ED diagnoses but be aware of its limitations.

### 4.1. Limitations

The study was limited by incomplete demographic information in the Victorian Emergency Minimum Dataset. The ED coding system may have also resulted in inaccuracies as it required ED doctors to search for specific keywords relating to their intended diagnosis. Coding inaccuracies may have resulted from inaccurate or broad keyword searches. Coding unavailability, such as an appropriate ICD-10 code for chronic ulcers, also resulted in the inappropriate classification of some dermatological conditions which may have concealed their true prevalence.

## 5. Conclusion

The burden of dermatological presentations to EDs in Australia may be somewhat larger than previously thought but overall remains small compared to other conditions. Cellulitis, allergy with skin involvement, and boils/furuncles/pilonidal sinuses were the most common presenting conditions. It is important that junior doctors receive appropriate training in the initial investigation and management of these common dermatological presentations. The study will have implications for curriculum design for ED doctors as well as inform them of decisions regarding dermatological resource allocation to ED and highlight the importance of an accessible dermatology consulting service via teledermatology.

## Supplementary Material

Appendix A: Keywords used to select dermatological presentations from the ‘Presenting Complaint' and ‘Triage notes' fields of the VEMD.
Appendix B: Selected emergency ICD10 codes used to select dermatological presentations to ED.

## Figures and Tables

**Table 1:** Demographic data for dermatological presentations compared to nondermatological presentations to ED.

	Dermatological presentations	Other ED presentations	*P* value
Age (years) (mean, median)	44.2	47.8	<0.001
	39.0	44.0	
Triage category 1	6 (0.1%)°	1245 (1.1%)*	<0.001
Triage category 2	187 (3.9%)°	9837 (8.3%)*	
Triage category 3	778 (16.2%)°	45140 (38.2%)*	
Triage category 4	3261 (67.7%)*	53646 (45.4%)°	
Triage category 5	585 (12.1%)*	8193 (6.9%)°	
Time to ED doctor (min) (median, IQR)	56.0	31.0	<0.001
	19–117	8–82	

*Overrepresented according to adjusted standardised residuals.

°Underrepresented according to adjusted standardised residuals.

**Table 2:** Top 20 skin complaints seen at SVHM ED by ICD-10 diagnostic code.

Complaint	*N *	% of total cases
Cellulitis	1741	36.1
Allergy with skin involvement	939	19.5
Boils/ furuncles/ pilonidal sinuses	536	11.1
Eczema/dermatitis	274	5.7
Varicella zoster	161	3.3
Nonspecific skin infections	135	2.8
Unlisted	109	2.3
Nonspecific viral infections with rash	98	2.0
Skin lump unspecified	82	1.7
Nonspecific rashes	79	1.6
Sebaceous cyst	71	1.5
Impetigo	69	1.4
Drug rash	55	1.1
Psoriasis	46	1.0
Superficial thrombosis/thrombophlebitis	41	0.9
Ingrown nail of finger/toe	37	0.8
*Candida* infections	31	0.6
Skin neoplasms	30	0.6
Pruritus	30	0.6
Scabies	27	0.6
