# Supplementary material for: Which Dermatological Conditions Present to an Emergency Department in Australia?
Source: Emerg Med Int. 2014 Mar 31;2014:463026. doi: 10.1155/2014/463026 (PMC3988721; doi:10.1155/2014/463026)
Supplement: Supplementary file 1 — Appendix A: Keywords used to select dermatological presentations from the ‘Presenting Complaint' and ‘Triage notes' fields of the VEMD. Appendix B: Selected emergency ICD10 codes used to select dermatological presentations to ED. [file 463026.f1.pdf]

Appendix A:
Keywords used to select dermatological presentations from the 'Presenting Complaint' and 'Triage notes' fields of the VEMD.

- Urticaria, angioedema, flushing, induration, hives
- Skin infections, cellulitis, impetigo, herpes, abscess, furuncle, carbuncle, varicella, chickenpox, hand foot and mouth, viral exanthum, warts, syphilis, candidiasis, erysipelas, scabies, measles, mumps, rubella, folliculitis, pseudofolliculitis
- Rash, erythema, maculopapular
- Eczema, dermatitis, lichen simplex chronicus
- Skin reactions, drug reaction, allergy
- Lymphadenitis
- Granulomas
- Myxoedema
- Prurigo
- Bites, stings, itch
- Vesicle, blister
- Ulcer
- Frostbite
- Psoriasis
- Behcet's
- Bullous, pemphigus, pemphigoid
- Lupus
- Pityriasis
- Steven Johnson syndrome
- Nail
- Sweat glands
- Seborrhoea/ seborrheic
- Mucosal rash/ ulcer
- Acne
- Skin cancer, melanoma
- Cyst
- Sunburn
- Corn
- Bunion

Appendix B:
Selected emergency ICD10 codes used to select dermatological presentations to ED

ICD-10-AM 7th	Full ICD-10-AM 7th edition description	VEMD description
A630	Anogenital (venereal) warts	Genital warts
A6300	Anogenital (venereal) warts, unspecified site	Anogenital (venereal) warts, unspecified site
A6301	Perianal (venereal) warts	Perianal (venereal) warts
A6302	Cervical (venereal) warts	Cervical (venereal) warts
A6303	Urethral (venereal) warts	Urethral (venereal) warts
A6304	Vaginal (venereal) warts	Vaginal (venereal) warts
A6305	Vulval (venereal) warts	Vulval (venereal) warts
A6306	Penile (venereal) warts	Penile (venereal) warts
A6307	Scrotal (venereal) warts	Scrotal (venereal) warts
A6309	Anogenital (venereal) warts, other site	Anogenital (venereal) warts, other site
B000	Eczema herpeticum	Eczema herpeticum
B001	Herpesviral vesicular dermatitis	Cold sore / Herpesviral vesicular dermatitis
B019	Varicella without complication	Chickenpox / Varicella
B029	Zoster without complication	Herpes zoster infection (includes shingles)
B059	Measles without complication	Measles
B069	Rubella without complication	Rubella / German measles
B07	Viral warts	Viral warts
B081	Molluscum contagiosum	Molluscum contagiosum
B084	Enteroviral vesicular stomatitis with exanthem	Hand, foot and mouth disease
B09	Unspecified viral infection characterised by skin and mucous membrane lesions	Exanthema, viral
B269	Mumps without complication	Mumps / Parotitis
B279	Infectious mononucleosis, unspecified	Mononucleosis, infectious
B349	Viral infection, unspecified	Viral infection
B350	Tinea barbae and tinea capitis	Tinea capitis / Tinea barbae
B353	Tinea pedis	Fungal foot infection / Tinea pedis / Athlete's foot / Dermatophytosis of foot / Foot ringworm
B359	Dermatophytosis, unspecified	Ringworm / Dermatophytosis
B360	Pityriasis versicolor	Tinea versicolor
B373	Candidiasis of vulva and vagina (N77.1*)	Candidiasis, vulval or vagina / Vaginal thrush
B379	Candidiasis, unspecified	Candidiasis, NEC
B49	Unspecified mycosis	Fungal infection / Fungaemia / Mycosis
B839	Helminthiasis, unspecified	Worms / Helminthiasis
B852	Pediculosis, unspecified	Lice infestation / Pediculosis
B86	Scabies	Scabies
B89	Unspecified parasitic disease	Parasitic disease, unspecified
D179	Benign lipomatous neoplasm, unspecified	Lipoma
D1800	Haemangioma, unspecified site	Haemangioma / Lymphangioma
D485	Neoplasm of uncertain or unknown behaviour of skin	Neoplasm, skin
D690	Allergic purpura	Henoch-Schonlein purpura / Allergic purpura
I776	Arteritis, unspecified	Vasculitis
I800	Phlebitis and thrombophlebitis of superficial vessels of lower extremities	Superficial embolism/thrombosis/venous thrombophebitis of lower limb

I801	Phlebitis and thrombophlebitis of femoral vein	Femoropopliteal phlebitis
L010	Impetigo [any organism] [any site]	Impetigo
L029	Cutaneous abscess, furuncle and carbuncle, unspecified	Boil / Furunculosis / Abscess, skin, any site
L0301	Cellulitis of finger	Onychia, finger / Cellulitis, finger / Paronychia, finger
L0302	Cellulitis of toe	Onychia, toe / Cellulitis, toe / Paronychia, toe
L0311	Cellulitis of lower limb	Cellulitis, leg (excludes cellulitis of toe: L0302)
L039	Cellulitis, unspecified	Cellulitis, skin (excludes cellulitis of leg: L0311, toe: L0302 or finger: L0301)
L050	Pilonidal cyst with abscess	Pilonidal cyst, fistula or sinus with abscess
L059	Pilonidal cyst without abscess	Pilonidal cyst, fistula or sinus without abscess
L089	Local infection of skin and subcutaneous tissue, unspecified	Skin infection
L209	Atopic dermatitis, unspecified	Atopic eczema / Dermatitis
L219	Seborrhoeic dermatitis, unspecified	Dermatitis, seborrhoeic
L22	Diaper [napkin] dermatitis	Nappy rash
L258	Unspecified contact dermatitis due to other agents	Dermatitis, contact, due to other agents
L270	Generalised skin eruption due to drugs and medicaments	Rash, drug, generalised
L271	Localised skin eruption due to drugs and medicaments	Rash, drug, localized
L272	Dermatitis due to ingested food	Dermatitis due to ingested food
L299	Pruritus, unspecified	Pruritus
L309	Dermatitis, unspecified	Dermatitis / Eczema, NEC (includes allergic reaction to plants)
L409	Psoriasis, unspecified	Psoriasis
L42	Pityriasis rosea	Pityriasis rosea
L509	Urticaria, unspecified	Urticaria
L519	Erythema multiforme, unspecified	Erythema multiforme
L539	Erythematous condition, unspecified	Erythema
L559	Sunburn, unspecified	Sunburn Unspecified
L600	Ingrowing nail	Ingrowing nail of finger or toe
L659	Nonscarring hair loss, unspecified	Alopecia
L721	Trichilemmal cyst	Sebaceous cyst
L740	Miliaria rubra	Heat rash
L899	Decubitus ulcer and pressure area, unspecified	Bedsore / Decubitus ulcer
L989	Disorder of skin and subcutaneous tissue, unspecified	Skin or subcutaneous tissue disorder NOS
M329	Systemic lupus erythematosus, unspecified	Systemic lupus erythematosus
M359	Systemic involvement of connective tissue, unspecified	Other diffuse connective tissue disorder
M7999	Unspecified soft tissue disorder, site unspecified	Soft tissue disorder unspecified
N750	Cyst of Bartholin's gland	Cyst of Bartholin's gland
N751	Abscess of Bartholin's gland	Abscess of Bartholin's gland
N760	Acute vaginitis	Vaginal infection
N762	Acute vulvitis	Vulval infection

N764	Abscess of vulva	Abscess of labia or vulva
R21	Rash and other nonspecific skin eruption	Rash, non-vesicular (excludes Nappy rash: L22, Urticarial rash: L509)
R229	Localised swelling, mass and lump, unspecified	Skin lump (excludes Breast lump: N63, Neck lump: R221)
R233	Spontaneous ecchymoses	Ecchymoses, spontaneous / Petechiae
R238	Other and unspecified skin changes	Seborrhoea
T780	Anaphylactic shock due to adverse food reaction	Anaphylactic shock, due to adverse food reaction
T781	Other adverse food reactions, not elsewhere classified	Reaction/Allergy to food (other than dermatitis)
T782	Anaphylactic shock, unspecified	Anaphylactic shock (excludes due to Vaccine: T805 or Food: T780)
T783	Angioneurotic oedema	Angioneurotic oedema / Giant urticaria / Quicke's oedema
T784	Allergy, unspecified	Allergy, NEC (excludes to Food: T781, Plants: T887, Vaccine: T806)
T805	Anaphylactic shock due to serum	Anaphylactic shock due to serum/vaccine/immunisation
T886	Anaphylactic shock due to adverse effect of correct drug or medicament properly administered	Anaphylactic shock, due to adverse effect of correct substance properly administered
T887	Unspecified adverse effect of drug or medicament	Reaction to drug, unspecified (excludes anaphylactic shock due to drug: T886)
